# Increasing number of organ dysfunctions is an excellent predictor of in-hospital mortality in emergency department patients with suspected infection: an internal and external prospective validation study

**DOI:** 10.1186/cc12924

**Published:** 2013-11-05

**Authors:** Marie K Jessen, Simon Skibsted, Nathan I Shapiro

**Affiliations:** 1 Research Center for Emergency Medicine, Aarhus University Hospital, Aarhus, Denmark; 2 Research Department of Emergency Medicine, Beth Israel Deaconess Medical Center and Harvard Medical School, Boston, MA, USA

## Background

Conscious assessment for organ dysfunction in infected patients is not uniformly performed since the prognostic performance of organ dysfunction has not been validated. We hypothesize that the number of organ dysfunctions is a prognostic marker in emergency department (ED) patients with suspected infection and that an increasing number of organ dysfunctions correlates with in-hospital mortality.

## Materials and methods

A prospective observational study of adult (18+ years) ED patients with suspected infection presenting to one of two urban, academic medical center EDs. The inclusion criterion was clinically suspected infection at ED presentation. At Beth Israel Deaconess Medical Center (BIDMC), Boston, USA, consecutive patients were enrolled over a 1-year period (internal validation set) and at Aarhus University Hospital (AUH), Aarhus, Denmark, a case-control study was performed (external validation set). Laboratory and clinical data were collected at enrollment to assess organ dysfunction. Primary outcome was in-hospital mortality. Logistic regression was performed to determine the independent mortality odds.

## Results

**Figure 1:**
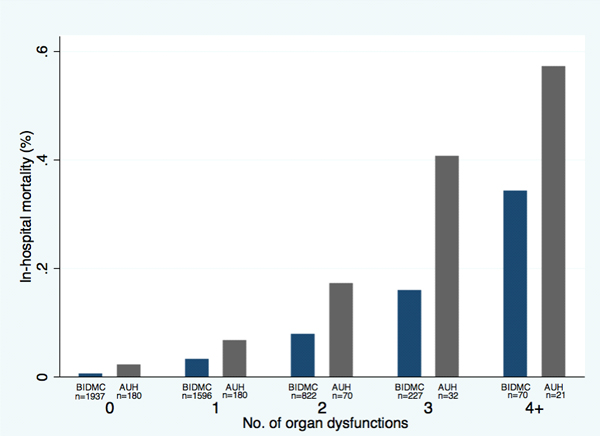


**Figure 2:**
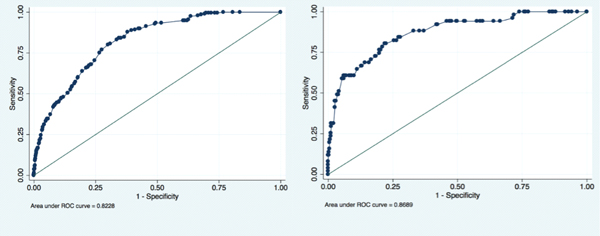


Four thousand, nine hundred and fifty-two patients were enrolled at BIDMC and 483 patients at AUH. Overall mortality rates were 4% and 11% with mean ages of 58 ± 21 and 69 ± 16 years, respectively. The mortality rate increased with increasing number of organ dysfunctions: BIDMC: 0 organ dysfunctions, 0.6% mortality; 1 dysfunction, 3.3%; 2 dysfunctions, 7.8%; 3 dysfunctions, 15.9%; and ≥4 dysfunctions, 34.3%; and AUH: 2.2%, 6.7%, 17%, 41%, and 57% mortality (Figure 1). The number of organ dysfunctions remained an independent predictor after adjustment for age and Charlson Index (Table 1). The AUCs for the models were 0.82 and 0.87, respectively (Figure 2). The effect of specific types of organ dysfunction on mortality was largest for respiratory dysfunction (OR 3.57 (95% CI 2.5 to 5.1)) in the internal and for hematologic dysfunction (OR 33.57 (8.56 to 127.3)) in the external validation set (Table 2).

**Table 1:** Effect of number of organ dysfunctions on in-hospital mortality adjusted for age and Charlson Comorbidity score

Number of organ dysfunctions	Internal validation set in-hospital mortality	External validation set in-hospital mortality
1	4.5 (2.3 to 8.6)	3.1 (0.9 to 10.4)
2	9.3 (4.8 to 18.1)	7.3 (2.1 to 24.7)
3	18.0 (8.8 to 36.9)	33.6 (8.56 to 127.3)
4	50.5 (22.0 to 115.8)	45.0 (8.56 to 236.2)
5	39.0 (8.9 to 170.7)	285.9 (16.9 to 483.2)

Data presented as OR (95% CI).

**Table 2:** Effect of organ dysfunctions on mortality adjusted for age and Charlson Index

Variable	Internal in-hospital mortality	External in-hospital mortality
Hematologic	3.4 (2.2 to 5.2)	29.0 (7.1 to 116.9)
Respiratory	3.6 (2.5 to 5.1)	1.4 (0.8 to 2.6)
Cardiovascular	3.2 (0.9 to 11.5)	18.5 (7.3 to 46.8)
Metabolic	3.0 (2.2 to 4.0)	6.7 (2.9 to 11.2)
Neurologic	2.4 (1.7 to 3.4)	8.9 (4.7 to 17.1)
Renal	2.1 (1.4 to 3.0)	5.4 (2.7 to 10.9)
Hepatologic	2.2 (1.4 to 3.4)	4.5 (1.1 to 18.2)

Data presented as OR (95% CI).

## Conclusions

Using readily available criteria in the ED to assess the number of organ dysfunctions is a reliable tool in predicting in-hospital mortality in both validation sets and could assist in risk prognostication and aid with earlier, targeted therapy.

